# Modeling reptile virus infection in vitro using *Python regius* airway organoids

**DOI:** 10.1038/s41467-026-76411-9

**Published:** 2026-08-13

**Authors:** Sam Willemsen, Gijs van Son, Melanie Rissmann, Ninouk Akkerman, Harry Begthel, Jeroen Korving, Carola Ammerlaan, Bart L. Haagmans, Johan van Es, Hans Clevers

**Affiliations:** 1 Hubrecht Institute, Royal Netherlands Academy of Arts and Sciences, Utrecht, The Netherlands University Medical Centre Utrecht, Utrecht, The Netherlands Oncode Institute, Utrecht, The Netherlands; 2 The Princess Máxima Center for Pediatric Oncology, Utrecht, The Netherlands; 3 Viroscience Department, Erasmus University Medical Centre, Rotterdam, The Netherlands

**Keywords:** Virology, Biological models

## Abstract

Zoonoses pose substantial global health risks, highlighting the need to better understand animal-to-human transmission. Reptiles are increasingly recognized as hosts of diverse pathogens, including numerous viruses, yet the diversity and prevalence of reptile pathogens, as well as their potential risk to humans, remain poorly understood. Here, we establish and characterize airway organoids derived from *Python regius*, providing an in vitro model to study reptile airway infection. Through de novo assembly of a *Python regius* reference genome, we characterize airway organoids at single-cell resolution, which suggests the presence of diverse cell populations including ionocytes, ciliated, secretory, goblet, endocrine, tuft, and basal cells. The organoids support productive infection with Ball Python Nidovirus (BPNV) and mount a robust epithelial antiviral response through the induction of interferon-stimulated genes, cytokines, and genes involved in chemical defense. As a proof-of-concept, treating organoids with antiviral drugs during infection reduces BPNV levels, highlighting the model’s utility for drug testing. By providing a reductionist system of the serpentes airway, these organoids constitute a physiologically relevant in vitro model to study reptile viruses and host–pathogen interactions in their native host.

## Abstract (web summary)

Ball python airway organoids, characterized at single-cell resolution, support productive infection with a reptile virus and respond to antiviral drugs, establishing a physiologically relevant model for studying reptile host–pathogen interactions.

## Introduction

It is estimated that approximately 75% of emerging infectious diseases in humans are zoonotic in origin^1^. Cross-species transmission of pathogens poses a substantial threat to global public health through their potential to cause epidemics or pandemics. Understanding the full diversity of pathogens, the range of animal hosts that harbor them, and the mechanisms and likelihood of cross-species transmission is therefore crucial. Despite major advances in our understanding of pathogens in their natural hosts, many species remain underexplored, largely due to the lack of suitable laboratory models or limited experimental access to the animal. Therefore, there has been a growing interest in the development of organoids for a range of animal species beyond conventional laboratory animals, especially those that act as important viral reservoirs^2^. Recent examples include organoids of bat airway, kidney, and intestine, avian intestine, bovine stomach, porcine airway, and camelid airway^3–7^.

Despite these advances in viral reservoir animal models, reptiles have received relatively little attention. Reptiles are known to be infected by a wide range of pathogens and have been recognized as reservoirs for numerous vector-borne diseases that are transmitted by arthropods that feed on both reptiles and humans^8^. Yet a substantial portion of reptilian pathogens remains uncharacterized, as our knowledge of them is not proportional to the abundance of their hosts^9–11^. Reptiles have also become popular exotic pets, and in some parts of the world serve as important sources of food, medicine, and materials, thereby accounting for an estimated ~20% of the value of all live animal trade^12^. Therefore, it is important to develop suitable models to study reptile-associated viruses, both to understand their diversity and to assess the potential risks they pose to animal and human health.

*Python regius* (*P. regius*), or Ball Python, is native to West and Central Africa. Its popularity as a companion animal brings it into frequent contact with humans. Although global numbers of the current pet population remain poorly documented, 3.9 million live *P. regius*, the vast majority of which were wild-caught, were traded between 1975 and 2018. This makes it the most exported CITES-listed live animal from Africa, accounting for 63% of the global live snake trade^13,14^.

Ball Python Nidovirus (BPNV) is a *serpentovirus* in the order of *Nidovirales* that most commonly infects *P. regius* but has also been documented in other species of snakes, lizards, and turtles^15,16^. BPNV causes respiratory disease with excessive oral mucus secretion followed by oral swelling, excessive swallowing, and anorexia, as well as an increased respiratory effort^17^. Treatment of captive snakes is limited to supportive care and eventual euthanasia^18^. Furthermore, subclinical infections can occur, turning animals into hidden reservoirs that may facilitate the spread of the virus through the live animal trade^17^. Serpentoviruses, including BPNV, represent a threat to captive snakes, as they spread rapidly and are associated with high morbidity and mortality^19^. In a recent assessment of roughly 1500 captive snakes, including animals with and without respiratory disease symptoms, roughly 28% tested positive for BPNV^18^. This high prevalence, combined with subclinical infections and the volume of snakes globally traded, underscores the risk of virus transmission in both wild and captive snake populations and, potentially, of human exposure. Indeed, others have recently highlighted the zoonotic potential of pathogens carried by exotic pets^10,20^.

While detailed molecular descriptions of the serpentine respiratory system remain limited, previous work has extensively described gross anatomical and microscopic features. The airway of most snakes consists of a trachea that first transitions into a bronchus and subsequently into a functional region of unicameral vascular lung^21^. The lung then terminates in an avascular, nonrespiratory air sac. Within the functional region, the microscopic organization consists of a honeycomb-like appearance of trabeculae (ediculae), covering smaller structures known as faveolae^22^. Faveoli are considered homologous to the mammalian alveoli, as this is where most gas exchange takes place. The faveolae are made up mostly of AT1 cells (which mediate O2 and CO2 exchange) with a few AT2 cells (responsible for surfactant production). The overlying epithelium of the ediculae is composed mostly of pseudostratified ciliated cells and lacks secretory cells, whereas the upper airway epithelium consists of basal, ciliated and secretory cells^23^.

Despite their importance, reptile viruses such as BPNV remain understudied. Suitable in vitro models, especially those representing target organs, are instrumental in facilitating research into these pathogens. Here, we apply organoid technology to develop an in vitro experimental platform for *P.* *regius* airway infection.

## Results

### Establishment of *P. regius* airway organoids

**Fig. 1:**
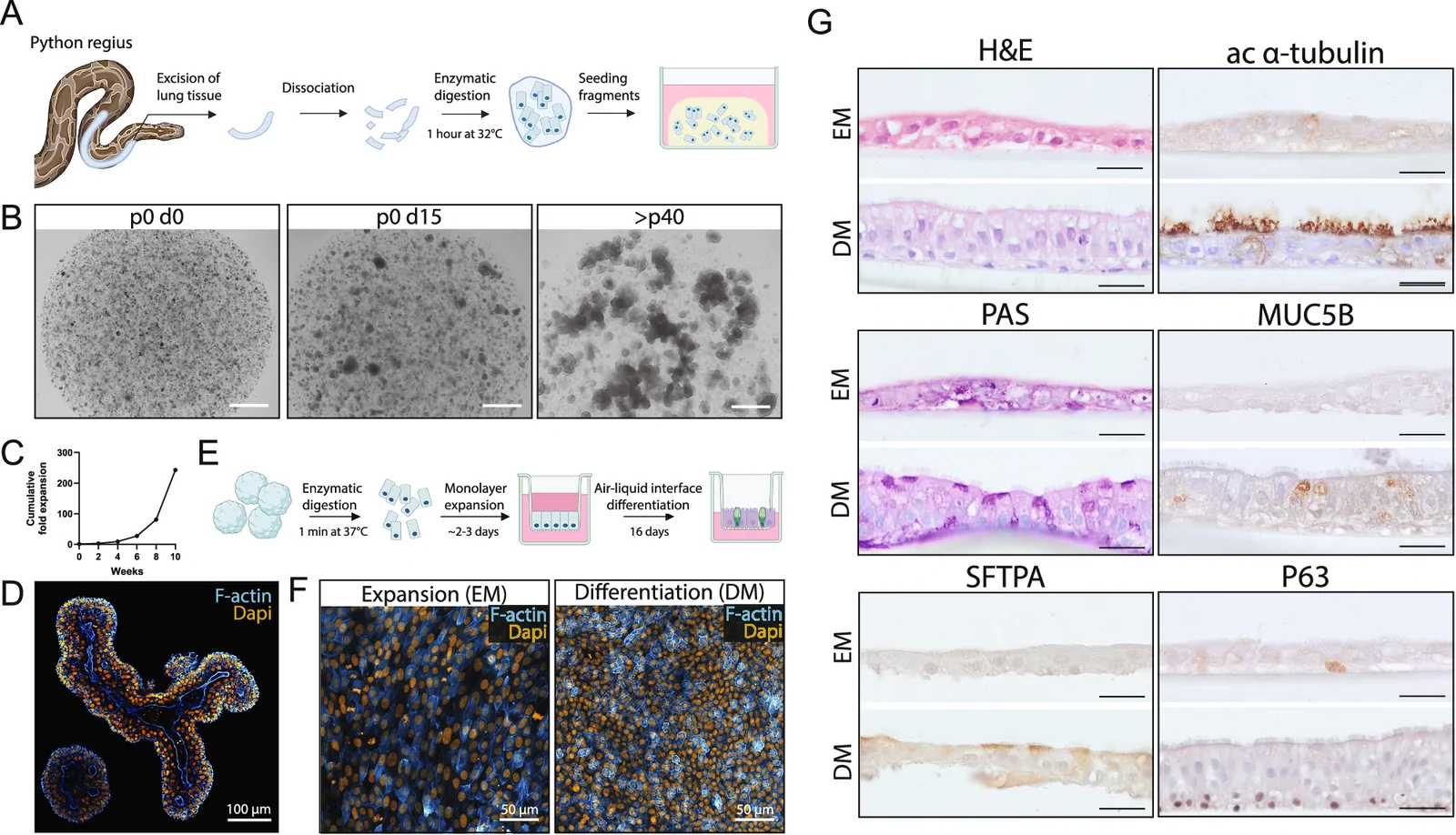
Establishment of *P. regius* organoids. **A** Graphical representation of the protocol for establishing *P. regius* airway organoids. **B** Representative brightfield images of fragments seeded from tissue on day 0 (left), initial organoid formation (middle), and organoids 10 days after passaging once the culture system was established (right). Representative images of one independent experiment. Scale bar = 500 µm. **C** The expected cumulative fold expansion of the organoids if passaged at a 1:3 split ratio every 2 weeks. **D** Immunofluorescence stainings of DAPI and F-actin highlighting different organoid morphology during expansion conditions. Representative images of 3 independent experiments. **E** Graphical representation of the differentiation protocol. **F** Immunofluorescence stainings of DAPI and F-actin of organoids grown in 2D in expansion medium at confluency (left) and after differentiation (right). Representative images of two independent experiments. **G** Histological characterization of organoids in expansion medium at confluency (EM) and differentiated organoids (DM). Representative images of three independent experiments. Scale bar = 20 µm. Panel A and E of Fig. 1 was created in BioRender. van Es, J. (2026) https://BioRender.com/r54n211.

To generate adult stem cell-derived organoids from the *P. regius* airway, the vascularized region of the right lung was excised and enzymatically digested into tissue fragments (Fig. 1A). Initial organoid formation occurred after roughly three weeks and organoid expansion persisted through at least 40 passages without signs of reduced growth potential (Fig. 1B). Organoids were established by directly seeding tissue fragments in basement membrane extract (BME) and culturing them in airway media, containing Noggin, R-spondin, and FGF7^24^. Once established, organoids were passaged in a 1:3 split ratio every 1-2 weeks (Fig.1C). During expansion, organoids grew as dense spheres gradually acquiring irregular shapes and forming branching structures with increasing size (Fig. 1D). For differentiation, organoids were seeded as monolayers and upon reaching confluency were cultured using commercial air-liquid interface medium (Fig. 1E). Organoid cells in 2D remained flat when cultured in expansion medium (EM), whereas air–liquid interface differentiation (DM) led to a more tightly packed and more pronounced pseudostratified epithelium (Fig. 1F, G). Furthermore, differentiated organoids displayed histological features of ciliated cells (acetylated α-tubulin), goblet cells (PAS and MUC5B), secretory cells (SFTPA) and basal cells (p63) (Fig. 1G). In summary, organoids exhibited robust expansion and, upon differentiation, formed a pseudostratified epithelium that contained cell types characteristic of the upper airway epithelium.

### Assembly of *P. regius* reference genome

Characterization of our organoids was challenging due to the paucity of validated antibodies and reagents and the lack of an annotated genome for *P. regius*. To this end, we established a *P. regius* reference genome (Supplementary Fig. 1A). The genome was constructed using short and long reads, utilizing the hybrid assembly method WENGAN (v0.2)^25^. Candidate genes were annotated by BlastP using the full non-redundant eukaryotic protein database from NCBI as a reference. BlastP annotation hits for identified gene transcripts can be found in Supplementary data 1. The resulting *P. regius* reference genome was 1.37 GB in length and included successful annotations for 11.404 unique genes (Supplementary Fig. 1B). The assembly was screened for contamination using the BlobToolKit and assessed for completeness using BUSCO^26,27^. Most of the sequences originated from the Chordata phylum with only a very low amount of contamination (Supplementary Fig. 1C, D). Using the eukaryote odb10 set from BUSCO, a completeness of 84% was observed for the assembly (Supplementary Fig. 1E). The assembly had a total of 28.763 contigs, with the longest being over 600 Kb, a N50 length of 84 Kb, and a L50 of 4777 contigs (Supplementary Fig. 1F). Using this reference genome, RNA sequencing data was mapped with high efficiency, showing mapping rates between 60 and 70% across both bulk and single-cell sequencing data (Supplementary Fig. 1G, H).

### *P. regius* airway organoids contain diverse cell types

**Fig. 2:**
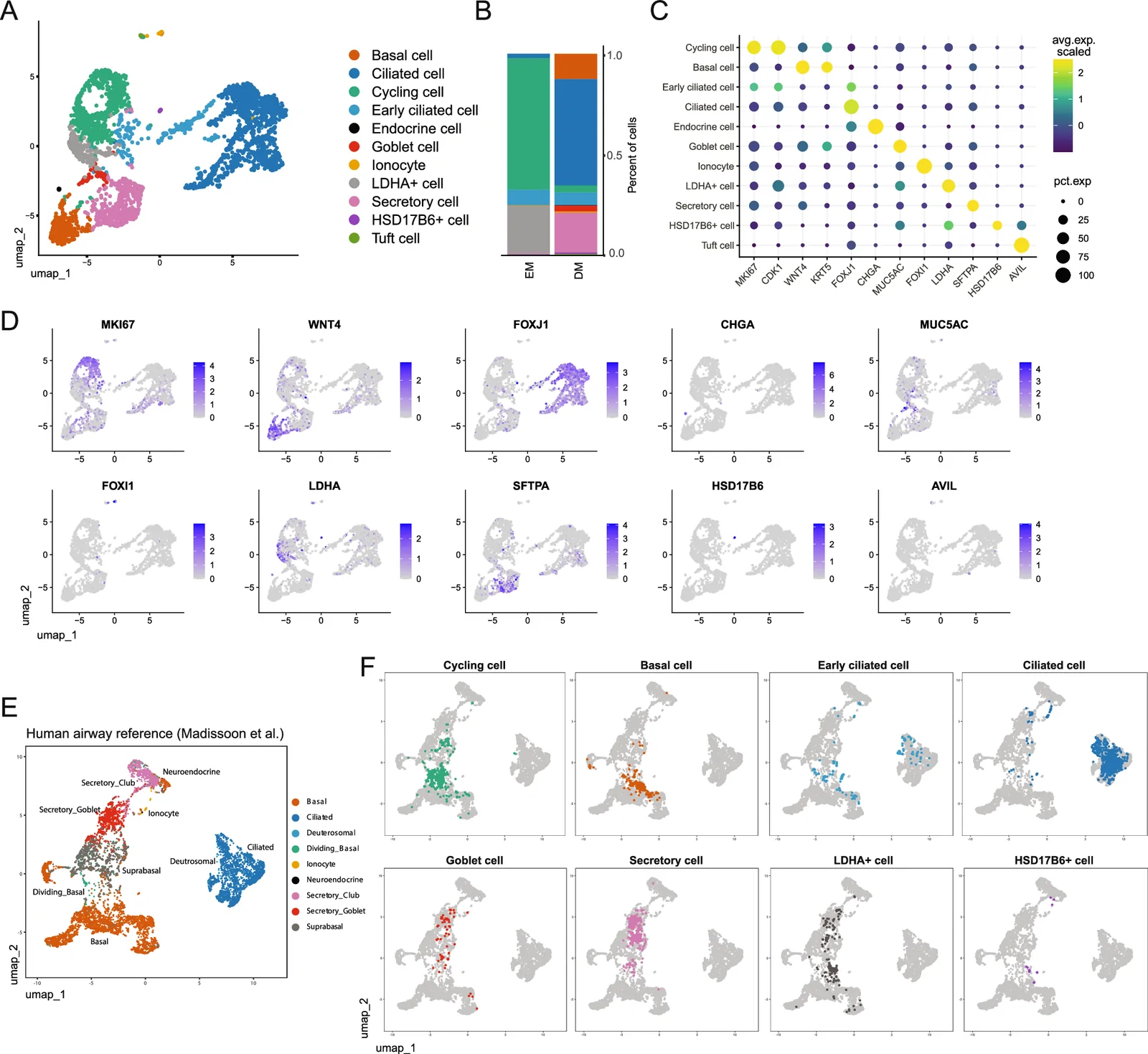
P. regius airway organoids contain diverse cell types. A) Uniform manifold approximation and projection (UMAP) of identified cell types in *P. regius* airway organoids. B) Relative contribution of each cell type population to organoids in expansion conditions (EM) and after differentiation (DM). C) Dot plot showing the average expression of marker genes for each cell cluster. D) UMAP showing the expression of marker genes for each cell cluster. E) UMAP of primary human data set, annotated for cell types. F) UMAP projection of each *P. regius* cell type (colored) onto the primary human data set (gray).

To evaluate the cellular diversity of our organoids, we performed single-cell RNA sequencing on organoids in EM and on organoids in DM. Unsupervised clustering and subsequent annotation based on known marker genes revealed 11 distinct airway cell populations (Fig. 2A). Organoids in EM contained mostly cycling cells along with small populations of early and late ciliated cells (Fig. 2B). Furthermore, a substantial number of cells in EM comprised of a population characterized by high expression of Lactate dehydrogenase A (LDHA), a population that was absent following differentiation. Differential gene expression analysis revealed additional markers for these LDHA+ cells, including OLFM4, IL17RB, FTH1, and VEGFA, but no clear functional interpretation (Supplementary Fig. 2A). Differentiated organoids consisted mostly of more mature cell types, as identified based on previously known marker genes (Fig. 2C, D). Based on the expression of key marker genes, we identified major mature airway cell types such as endocrine cells (CHGA^+^), ionocytes (FOXI1^+^), tuft cells (AVIL^+^), mucus-producing goblet cells (MUC5AC^+^), surfactant A-producing secretory cells (SFTPA^+^), and early (FOXJ1^Mid^) and late ciliated cells (FOXJ1^High^). Differentiation also led to the appearance of Basal cells (WNT4 + , KRT5 + ), and a small fraction of the actively cycling cells persisted from the EM condition. Furthermore, a small population of cells could not be annotated based on a clear signature but was strongly enriched for alcohol oxidoreductase HSD17B6. Differential gene expression analysis revealed additional markers for HSD17B6+ cells, including TAT, CLEC10A, KNG1, AMBP, ITIH4, and TDO2, but no clear functional interpretation (Supplementary Fig. 2A). Next, we integrated our *P. regius* organoid data with a primary tissue dataset. Since no single-cell RNA sequencing dataset was available for snake airways, we instead used an extensively annotated primary human airway cells atlas (Fig. 2E and Supplementary Fig. 2B)^28^. Projection of *P. regius* organoid data onto primary upper airway epithelium data revealed that cycling cells, basal cells, ciliated cells, ionocytes, and goblet cells clustered with their human counterparts (Fig. 2F and Supplementary Fig. 2C). SFTPA+ secretory cells did not directly cluster with human secretory club cells, suggesting that they are not mature club cells. Since SFTPA+ cells did not express any mucus genes or club cell markers, yet clustered near human secretory mucus cells, they might represent an immature stage of the secretory lineage. LDHA+ cells overlapped with cycling, basal, and secretory cells, further suggesting that they may represent an intermediate, transient population during airway epithelium differentiation. To broaden our comparative analysis, we also integrated our organoids with a single-cell atlas of 11 non-model animals^29^. Based on the annotation of the atlas, *P. regius* cells clustered away from AT1 and AT2 cells, suggesting our organoids do not represent the faveolae (snake alveolae), while secretory and ciliated cells clustered with their corresponding cell types across all different species (Supplementary Fig. 2D). Together, these findings provide a comprehensive molecular atlas of *P. regius* airway organoids, revealing a broad diversity of cell types.

### Ball Python Nidovirus (BPNV) replicates in *P. regius* airway organoids and elicits an epithelial immune response

**Fig. 3:**
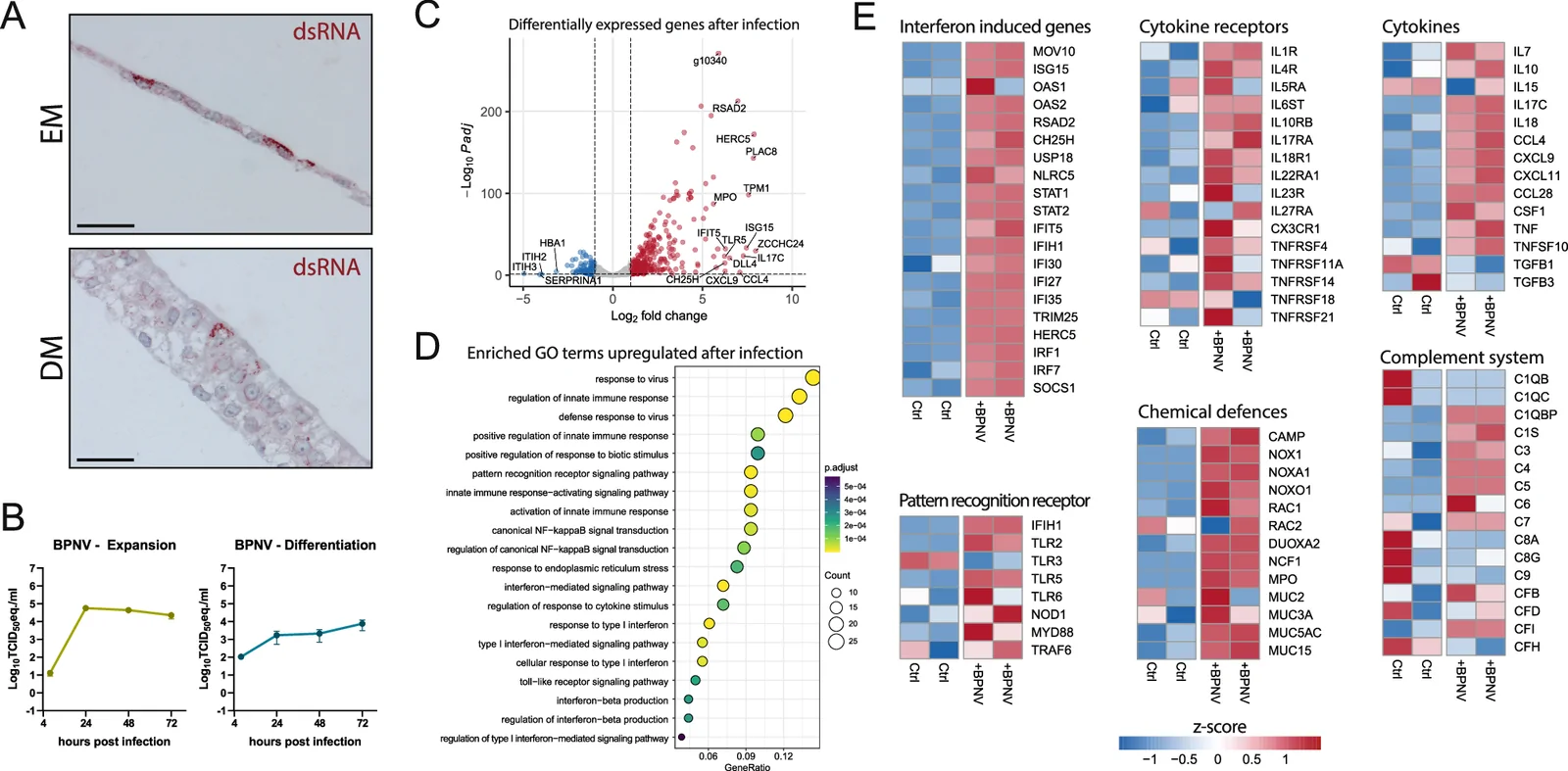
Ball python nidovirus (BPNV) replicates in *P. regius* airway organoids and elicits an epithelial immune response. **A** Staining of dsRNA in infected expansion (EM) or differentiated (DM) 2D organoids after 72 h. Representative images of two independent experiments. Scale bar = 20 µm. **B** Quantification of BPNV genome levels by qPCR after infection in expansion or differentiated 2D organoids. Data are presented as mean values ± SD, *n* = 3 technical replicates per condition. TCID_50_eq = Tissue Culture Infectious Dose 50% equivalents. **C** Volcano plot showing the 15 highest upregulated and downregulated differentially expressed genes after infection of differentiated 2D organoids. **D** Enriched GO terms on 2logFC upregulated genes after infection of differentiated 2D organoids. **E** Heatmaps highlighting the upregulated genes from key host defense pathways. Shown are genes annotated in the *P. regius* genome; pathway-relevant genes missing from the annotation are not shown.

To determine whether *P. regius* organoids could serve as an in vitro model for studying viral infection, we infected differentiated organoids with BPNV, a double-stranded RNA (dsRNA) *serpentovirus* in the order of *Nidovirales*. This virus most commonly infects *P. regius*  and causes respiratory symptoms. Organoids were differentiated as a monolayer and were exposed to BPNV from the apical side. BPNV was able to effectively replicate in *P. regius* airway organoids in EM and DM (Fig. 3A). At early time points, organoids in EM showed higher viral genome levels than those in DM, with similar levels at later time points (Fig. 3B). As a control, organoids were also infected with avian-adapted influenza H5N1, which failed to replicate productively (Supplementary Fig. 3A). After BPNV infection, cytopathic effects were observed 48 h post-infection, but epithelial monolayers remained viable (Supplementary Fig. 3B). To assess the snake epithelial immune response to viral infection, we performed bulk RNA sequencing of differentiated organoids with and without BPNV exposure. Among upregulated genes with the highest fold changes were interferon-stimulated genes RSAD2, ISG15, IFIT5, and HERC5, as well as pro-inflammatory genes CCL4, CXCL19, and IL17C (Fig. 3C). GO term enrichment analysis against human GO categories for genes upregulated by at least 2 logFC revealed terms related to antiviral response, interferon signaling, innate immunity and pattern recognition receptor signaling, suggesting that the *P. regius* organoids consisted of an immunologically responsive airway epithelium (Fig. 3D). There were no enriched GO terms based on downregulated genes. We then assessed the effect of BPNV on the expression of a large number of known genes from key host defense pathways (Fig. 3E). The *Python regius* genome did not have all pathway-relevant genes annotated, which were unable to be assessed as a result. Type I and type II interferons were amongst these genes without annotation. However, a large number of interferon-stimulated genes were upregulated after infection with BPNV, nevertheless hinting at active interferon signaling or a similar antiviral response. The epithelium did show an increased expression of other cytokines, including interleukins, chemokines, and members of the tumor necrosis factor and transforming growth factor beta families, as well as cytokine receptors. Different pattern recognition receptors (PRRs) were also expressed and upregulated after BPNV infection, with the notable exception of TLR3, a receptor for double-stranded RNA, which was downregulated. Additionally, the epithelium exhibited an active innate chemical immune response, as evidenced by the induction of cathelicidin antimicrobial peptide (CAMP), and ROS-producing genes NOX1, NOXA1, NOXO1, RAC1/2, and DUOXA2, as well as ROS-associated genes NCF1 and MPO, which are more commonly found in immune cells as opposed to epithelial cells. Finally, multiple mucin genes were upregulated, including respiratory tract-associated MUC5AC, reflecting a hallmark in vivo symptom of BPNV, excess mucus production, in the organoids after infection. These results demonstrated that our airway organoids supported BPNV replication in vitro and could elicit a robust antiviral and inflammatory epithelial response upon infection.

### *P. regius* organoids as a physiologically relevant in vitro model for antiviral drug testing

**Fig. 4:**
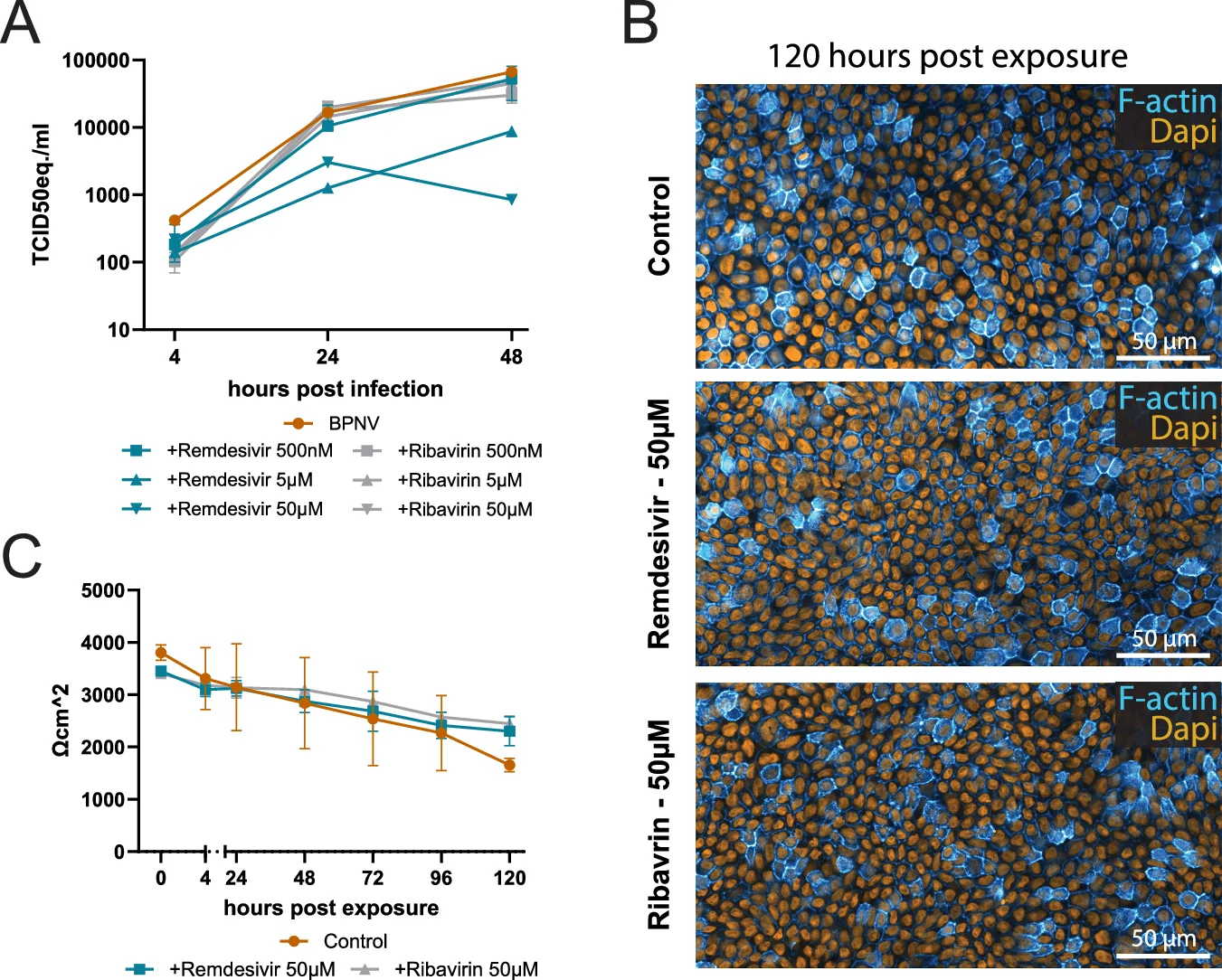
*P. regius* organoids as a BPNV drug testing platform. **A** Quantification of BPNV genome levels by qPCR in 2D differentiated 2D organoids treated with different concentrations of antiviral drugs at the time of infection. Data are presented as mean values ± SD, *n* = 2 technical replicates per treatment. TCID_50_eq = Tissue Culture Infectious Dose 50% equivalents. **B** Immunofluorescence stainings of DAPI and F-actin of untreated 2D organoids and 2D organoids treated with the highest dose of Remdesivir or Ribavirin after 120 h. Representative images of two independent experiments. **C** Trans-epithelial electrical resistance (TEER) of untreated 2D organoids and 2D organoids treated with the highest dose of Remdesivir or Ribavirin for different time points. Data are presented as mean values ± SD, *n* = 4 technical replicates per condition.

Current treatments for BPNV infections are limited to supportive care and euthanasia. Since the organoids recapitulate key characteristics of the airway epithelium, we performed a proof-of-concept experiment to explore whether they could be leveraged to test antiviral drugs. Ribavirin and Remdesivir are broad-spectrum antivirals that have been previously reported to be effective against BPNV in experimentally infected primary Diamond python heart cells^30^. In our airway organoids, Remdesivir but not Ribavirin was able to reduce viral loads upon administration during BPNV infection (Fig. 4A). Importantly, the highest dosage of antivirals tested did not affect overall cellular morphology (Fig. 4B) nor did they impact monolayer integrity, even at later timepoints beyond the original experiment’s duration (Fig. 4C). Thus, *P. regius* airway organoids may represent a more physiologically relevant model for drug response testing.

## Discussion

To our knowledge, our *P. regius* airway organoids represent the first reptilian airway organoid model. Through de novo assembly of the *P. regius* reference genome, we were able to perform detailed molecular characterization of the *P. regius* airway organoids using both bulk- and single-cell RNA sequencing. These organoids recapitulated key features of airway epithelial cells in terms of complexity and diversity and, importantly, could mount a robust epithelial immune response upon BPNV infection. This organoid model is a physiologically relevant in vitro system for investigating understudied reptile viral pathogens in the context of their natural host species.

*P. regius* organoids showed a broader diversity of cell types than previously documented in the snake airway, including actively cycling cells, basal cells, ciliated cells, goblet cells, and secretory cells. Furthermore, the expression of key transcription factors might indicate the presence of rare cell populations such as endocrine cells, ionocytes, and tuft cells. The snake airway has previously been reported to contain endocrine, ciliated, and secretory cells in the upper airway and the ediculae, and AT1- and AT2-like cells in the faveoli, the reptilian equivalent of mammalian alveoli^23^. The absence of annotations of AT1 and AT2 marker genes in the *P. regius* genome, and their absence in our single-cell data, indicates that our organoids rather represent the upper airway and the ediculae. However, due to limited molecular characterization of these two regions in vivo, a clear distinction cannot be made. Furthermore, we describe a substantial population of LDHA⁺ cells, present only under expansion conditions, that overlaps with cycling, basal, and secretory cells and may represent an intermediate or transitional progenitor population during airway differentiation.

Our organoids supported replication of BPNV and could mount a robust innate immune response upon infection, making it a serpentine in vitro infection model derived from the in vivo target tissue of this virus. Previous studies experimentally infected *P. regius* with BPNV isolates via oral and intratracheal inoculations and described the infection at the organism level. These authors established a causal relation between BPNV infection and respiratory disease, describing mucinous chronic inflammation and proliferative pneumonia as hallmark pathological features^17^. Using organoids and bulk RNA sequencing, we molecularly characterized the epithelial immune response. Infected organoids exhibited increased expression of genes involved in mucus production and inflammation, reflecting in vivo symptoms. Differentially expressed genes also included interferon-stimulated genes RSAD2, ISG15, IFIT5, and HERC5. Gene Ontology Analysis, while limited due to reliance on human-based ortholog mappings, further highlighted terms related to antiviral response, interferon signaling, and innate immunity. Together with the high expression of cytokines and cytokine receptors in the epithelium, this indicated that P. *regius* airway organoids likely possess an anti-viral response and might be immunologically responsive. However, due to technical limitations, the *P. regius* genome lacked annotations for several relevant signaling pathway genes, limiting assessment of well-studied antiviral signaling and likely resulting in an incomplete representation of specific pathways. In summary, *P. regius* airway organoids represent a model to investigate virus–host interactions in a non-mammalian system.

These organoids represent an opportunity to investigate antivirals on BPNV and other serpentoviruses in the context of the natural host and in the target tissue. We treated organoids with two antivirals. Remdesivir reduced BPNV replication in *P. regius* airway organoids at 5 and 50 µM, whereas Ribavirin had no effect at any concentration tested. Notably, Remdesivir required higher concentrations than previously reported in Diamond python heart cells, suggesting reduced drug sensitivity in our organoids^30^. The authors showed potent antiviral activity of Remdesivir and Ribavirin against BPNV at EC_50_ values of 35 nM and 3.6 µM, respectively. This reduced compound sensitivity may be explained by the presence of a mucus layer on the apical surface of the organoids, which could hinder drug penetration and lower the effective concentration at the site of infection^31,32^. The airway is a common physiological target of BPNV, unlike cardiac cells, and therefore, this proof-of-concept experiment highlights the potential relevance of our organoid model for assessing antiviral efficacy in a more physiologically relevant tissue.

A limitation of our study is that our organoids were established from a single, near-hatchling specimen. Cell-intrinsic properties and cell-type composition are known to vary with age^33,34^. As such, our model might reflect a juvenile stage of *P.* *regius* airway biology, which may differ from adult airway physiology. This developmental specificity and lack of inter-individual variability should be considered when extrapolating our findings.

While *P. regius* can be studied under laboratory conditions, our organoid system reduces reliance on these long-lived animals, which have a captive lifespan of 20–30 years, and enables larger-scale veterinary research through a more accessible in vitro alternative. Furthermore, not all viral pathogens are easily propagated in vitro. For example, human noroviruses can only be efficiently propagated in organoids derived from the human small intestine, whereas conventional cell lines do not support this^35^. Similarly, *P. regius* airway organoids may potentially facilitate the in vitro propagation of a broader range of reptile pathogens.

In summary, we established and characterized *P. regius* airway organoids and demonstrated that they recapitulate key aspects of the in vivo airway, support BPNV infection, mount a robust epithelial antiviral response, and serve as a more physiologically relevant platform for testing antiviral compounds. These organoids can be expanded indefinitely and, by offering a reductionist model of the in vivo airway, provide a more accessible system for studying snake airway biology and for propagating BPNV and potentially other reptile viruses in vitro. This work has potential implications for the treatment of reptile pets, serpent ecology, and research on zoonotic diseases.

## Methods

### Organoid culture

A captive-bred *P. regius* egg was purchased in accordance with relevant regulations from a licensed reptile breeder. Ethical review was not required because fetal snakes (still inside the egg) are not classified as experimental animals under European and Dutch legislation prior to hatching. Please refer to the “European directive on the protection of animals used for scientific purposes” (Article 1, point 3)^36^. A few days prior to the expected hatching date, the snake was removed from the egg via an incision in the shell and euthanized via severing of the spinal cord at the base of the skull. A transverse incision was made along the length of the body. Using surgical scissors, a roughly 2 cm section of the vascularized region of the right lung was removed and minced into ~1 mm two pieces. Tissue pieces were resuspended in PBS and disrupted further by mechanical disruption with a 10 mL glass pipette and then left to settle by gravity. The resulting pellet was resuspended in 5 mL Advanced DMEM-F12 (ADF) with 1 mg/mL collagenase and incubated for 1 h at 32 °C under constant rocking. After digestion, the cells were passed through a 100μm cell strainer and washed twice with ADF. Cells were pelleted at 500 x g for 5 min, plated in basement membrane extract (BME; Cultrex) and incubated at 32 °C for 20 min before adding expansion media. The cells were cultured at 32 °C, and after initial organoid outgrowth after 3 weeks, they were passaged every 1-2 weeks. Passaging consisted of making organoids from single cells with a 1-minute incubation at 37 °C in cold TrypLE with 2.5 μM Rho-kinase inhibitor. Cells were washed twice with ADF to stop the digestion and spun down at 500 g for 5 min before seeding into BME at a 1:3 split ratio. For cryopreservation, organoids were collected when they reached sufficient size for passaging. Per cryovial, 200 µL BME with organoids was washed twice with ADF to remove BME. Organoids were then resuspended in 1 mL of Cellbanker 1 (Amsbio, 11910) and stored at −80 °C. When thawed, organoids were washed twice with ADF and plated in 200 µL BME. Airway organoid expansion medium (EM) consisted of: Advanced DMEM-F12 (ADF) (Thermo Fisher Scientific, 12634–010), 100U/ mL Penicillin-Streptomycin (Thermo Fisher Scientific, 15140122), 10 mM HEPES (Thermo Fisher Scientific. 15630080), 2 mM GlutaMAX (Thermo Fisher Scientific, 35050061), 1x Noggin-Fc fusion protein conditioned medium (IPA therapeutics, N002), 1x Rspo3-Fc fusion protein conditioned medium (IPA therapeutics, R001), 1x B-27 Supplement (Thermo Fisher Scientific, 17504044), 1.25mM N-Acetyl-L-cysteine (Sigma-Aldrich, A9165), 2.5 μM Y-27632 dihydrochloride (Rho-kinase inhibitor) (AbMole, M1817), 500 nM A83-01 (Tocris, 2939), 1 μM SB202190 (P38i) (Sigma-Aldrich, S7076), 25 ng/ mL hFGF-7 (Thermo Fisher Scientific, 100-19), 100 ng hFGF-10 (Thermo Fisher Scientific, 100-26) and 100 ng/ mL Primocin (InvivoGen, ant-pm-2).

### Organoid differentiation

Organoids were dissociated into single cells by incubating in cold TrypLE containing 2.5 μM Rho-kinase inhibitor at 37 °C for 1 min, followed by two washes with ADF to stop the digestion. Transwells (Greiner Bio-One, 662641) were precoated with 200 µL of 5% BME in ADF for 30 min at 37 °C. The BME solution was then aspirated, and the Transwells were incubated for an additional 30 min at 37 °C. For each Transwell, 200,000 single cells were plated in 200 µL of EM in the top compartment, and 500 µL of EM was added to the bottom compartment. After two days, the monolayers grew confluent. EM was then aspirated, and PneumaCult™ ALI Medium (STEMCELL technologies, 05001) was added to the bottom compartment while the top compartment was left empty. ALI medium was changed every 2–3 days for a duration of 16 days.

### Virus infection

Organoid monolayers at confluency in expansion medium or after differentiation were inoculated with 5000 viral particles in 200 µL ADF of either Ball Python Nidovirus (kindly provided by Dr. Mark Stenglein, Colorado State University, United States) or highly pathogenic avian influenza (HPAI) H5N1 virus (A/Indonesia/5/2005) (kindly provided by Dr. Lisa Bauer, Department of Viroscience, Erasmus Medical Center, The Netherlands). After an incubation for four hours at 32 °C, virus dilutions were removed, and cells were washed three times with ADF. Where applicable, antiviral drugs Remdesivir (MedChemExpress, HY-104077) and Ribavirin (MedChemExpress, HY-B0434) were added to the apical compartment of the cultures in described concentrations simultaneously with the virus dilution. Antiviral drugs were removed together with viral inoculum, and cells were washed three times with ADF.

### Virus quantification

Virus quantity was determined by qPCR at 4, 24, 48 and 72 h. For differentiated organoids, virus sampling was performed by adding and harvesting 200 μL of ADF at the apical side after a 20-minute incubation at 29 °C. For cells in expansion, 200 µL of ADF was sampled and replaced with fresh medium. Total RNA was isolated by lysing 60 μL of sample in 90 μL of MagnaPure LC Lysis buffer (Roche) followed by a 30-min incubation with 50 μL Agencourt AMPure XP beads (Beckman Colter). Beads were washed thrice with 70% ethanol on a DynaMag-96 magnet (Invitrogen) and eluted in 50 μL diethylpyrocarbonate-treated water. RNA extracted from BPNV-inoculated organoids was reverse transcribed into cDNA with SuperScript™ IV Reverse Transcriptase using Random Hexamer Primers according to the manufacturer’s protocol (Promega). qRT-PCR was performed using SYBR GREEN PCR Mastermix (Applied Biosystems) on a 7500 Real Time PCR Cycler (Applied Biosystems) and previously described primers and cycling conditions^17^. RNA extracted from H5N1-inoculated organoids was reverse transcribed into cDNA with TaqMan Fast Virus 1-Step Master Mix (Life Technologies) and a previously described primer–probe mix for IAV (FW: CTTCTRACCGAGGTCGAAACGTA, RV: TCTTGTCTTTAGCCAYTCCATGAG, Probe: 6-FAM TCAGGCCCCCTCAAAGCCGARA BHQ1)^37^. The qRT-PCR temperature profile was 5 min at 50 °C, 20 s at 95 °C, followed by 45 cycles of 3 s at 95 °C and 30 s at 60 °C. For quantification of H5N1 and BPNV-specific RNA, Ct values were compared to a standard curve derived from a titrated virus stock for each.

### Immunofluorescence and immunohistochemistry

Organoids cultured in 3D were collected from BME domes by mechanical disruption, washed three times with ADF to remove BME, and then fixed with 4% PFA for 30 min. Organoids were permeabilized for 30 min in 0.1% Triton, 0.2% BSA-PBS. Organoids were stained for F-actin with 1000x Phalloidin-Atto 647 N (Sigma, 65906-10NMOL) and 1000x DAPI at room temperature for 2 h. Organoid monolayers were washed with PBS twice and then fixed with 4% PFA for 30 min. Organoid monolayers were dehydrated, paraffin-embedded, and sectioned. Standard H&E and PAS stainings were performed as well as immunohistochemistry using antibodies against acetylated α-tubulin (1:2000; Santa Cruz, sc-23950), MUC5B (1:500; Santa Cruz, sc-21768), P63 (1:800; Abcam, ab735), and SFTPA (1:250; Abcam, ab325321). For the detection of BPNV, antigen was retrieved by incubating sections for 10 min in 0.1% protease at 37 °C. The slides were then washed with PBS/0.05% Tween 20 and incubated with mouse IgG2a-anti-dsRNA (J2; 1 mg/ml, #10010200 Nordic/MUbio) in PBS/0.1% BSA overnight at 4 °C. After washing, sections were incubated with biotinylated rabbit-anti-mouse Ig (#1010-08; Southern Biotech) in PBS/0.1% BSA for 1 h at RT. After washing, slides were incubated with a streptavidin-HRP conjugate (D0397; Dako) for 1 h at RT. Peroxidase activity was visualized with 3-amino-9-ethylcarbazole (AEC; Sigma-Aldrich) for 10 min, yielding a red precipitate, and counterstained with hematoxylin.

### TEER measurements

Trans-epithelial electrical resistance (TEER) was measured in differentiated organoid monolayers using the Millicell® ERS 3.0 Digital Voltohmmeter. After 16 days of differentiation, ADF alone or supplemented with 50 µM Ribavirin or Remdesivir was added apically, with medium refreshed daily in both compartments. During measurements, media were replaced with room-temperature ADF.

### De novo genome assembly

The *P. regius* reference genome was established by combining Illumina and Nanopore DNA sequencing for optimal coverage. For Illumina sequencing, DNA was extracted from pooled, snap-frozen primary tissue biopsies of stomach, intestine, and colon using a genomic DNA purification kit (ZYMO RESEARCH). DNA libraries were constructed using a TruSeq DNA nano library prep kit (Illumina). This library was subjected to paired-end sequencing (2 x 300 bp) on an Illumina NextSeq2000 machine. For Nanopore sequencing, DNA was isolated from a separate set of pooled, snap-frozen stomach, intestine, and colon biopsies using a Monarch HMW DNA extraction kit (T3060S). Libraries were constructed using a Ligation Sequencing kit V14 (Oxford Nanopore) and sequenced on a GridION ONT10000 machine (Oxford Nanopore). Fastq files were generated and used for genome assembly. The genome was constructed using short and long reads, utilizing the hybrid assembly method WENGAN (v0.2)^25^. The script describing the step-by-step genome construction is available through GitHub (see also Code availability statement). The transcriptome was constructed from bulk RNA-sequencing reads from *P. regius* primary airway tissue and organoids (see also methods “Bulk RNA sequencing”) and placed on the genome using BRAKER3 (v3.0.8)^38^. Genes were annotated using the full non-redundant eukaryotic protein database from NCBI as a reference. Genes were functionally annotated using BlastP. Transcripts without a confident blast hit retained their gene number (denoted as “g” followed by numbers) from the analysis. Where a blast hit could be found, the gene symbol was added to the annotation (see also Supplementary Data 1). Genome assembly and annotation quality benchmarking was performed using the BlobToolKit^26^. Genome coverage, blast hits (diamond and blast), and BUSCO scores (v6.1.0) were added to the assembly blob and plotted using the blobtools viewer (v4.4.1)^27^.

### Single-cell RNA sequencing

Organoids grown in expansion and differentiated organoids were made single-cell through a 1-minute incubation at 37 °C in cold TrypLE with 2.5 μM Rho-kinase inhibitor. Differentiated organoids were directly harvested from the transwells using TrypLE to minimize cell death. Single-cell samples were washed twice with ADF before proceeding with library preparation, performed according to standard protocols for 10 × 3’ sequencing using on-chip multiplexing on a GEM-X machine. The library was sequenced on an Illumina Novaseq6000 sequencer. Raw sequencing data were processed and mapped to our reference genome using Cellranger (v9.0.0). Downstream analysis was performed in R using Seurat. Briefly, unsupervised clustering resulted in 11 Seurat clusters. Cell identities were then attributed to cell clusters based on previously established marker genes. For the remaining unknown clusters, differential expression versus all other clusters was performed to identify marker genes. *P. regius* single-cell sequencing data were integrated with a human dataset by annotating *P. regius* genes by their closest human homolog. Python airway cells were subsequently projected onto a previously published annotated human airway single-cell dataset^28^. The dataset was subsetted to exclude single-nuclei sequencing data and to only include single-cell data from epithelial cells.

### Bulk RNA sequencing

Bulk RNA sequencing was performed on flash-frozen *P. regius* primary airway tissue, two replicates of differentiated airway organoids infected with BPNV, and two replicates of differentiated airway organoid controls. RNA was isolated from cells using the Nucleospin RNA kit (BIOKÉ). Organoid monolayers were harvested by scraping the transwell insert while pipetting lysis buffer up and down. Primary tissue was flash-frozen in TRIzol (Invitrogen) and stored at −80 °C immediately after dissection. The tissue was quickly thawed in a water bath and homogenized with a tissue homogenizer before RNA isolation according to the manufacturer’s instructions. Sequencing was performed at USEQ. Libraries were generated using the TruSeq RNA-stranded polyA library prep kit (Illumina). Libraries were subjected to paired-end sequencing (2 x 300bp) on an Illumina NextSeq 2000 machine. Reads were then mapped to the custom reference genome using STAR (v2.7.5c). Analysis was performed using a DESEQ2 (v 1.46.0)^39^. Differential expression was considered significant with a p-adjusted value below 0.05 and a baseline of 50 transcripts across all conditions. GO term analysis was performed using the R package clusterProfiler against the human database for GO biological pathways^40^.

### Reporting summary

Further information on research design is available in the Nature Portfolio Reporting Summary linked to this article.

## Supplementary information

Description of additional supplementary files

Supplementary information file

Supplementary data 1

Reporting Summary

Transparent Peer Review file

## Source data

Source Data

## Data Availability

The P. regius reference genome generated in this study is available on NCBI under bioproject number PRJNA1322132. The whole-genome sequencing data generated in this study are freely available in the SRA under accession numbers SRX31458866 and SRX31458867 for Illumina and Nanopore reads, respectively. The bulk RNA sequencing data generated in this study are available on GEO under accession number GSE313816; the processed data (count tables) are also provided there. The single-cell RNA sequencing data is available on GEO under accession number GSE313817, and count tables and metadata are also provided there. Source data are provided with this paper.

## Code availability

All scripts used for analysis are available on https://github.com/Hubrecht-Clevers/Python_regius_project^41^.
