# Supplementary material for: Modeling reptile virus infection in vitro using Python regius airway organoids
Source: Nat Commun. 2026 Aug 13;17:9739. doi: 10.1038/s41467-026-76411-9 (PMC13562644; doi:10.1038/s41467-026-76411-9)
Supplement: Supplementary file 1 [file 41467_2026_76411_MOESM2_ESM.pdf]

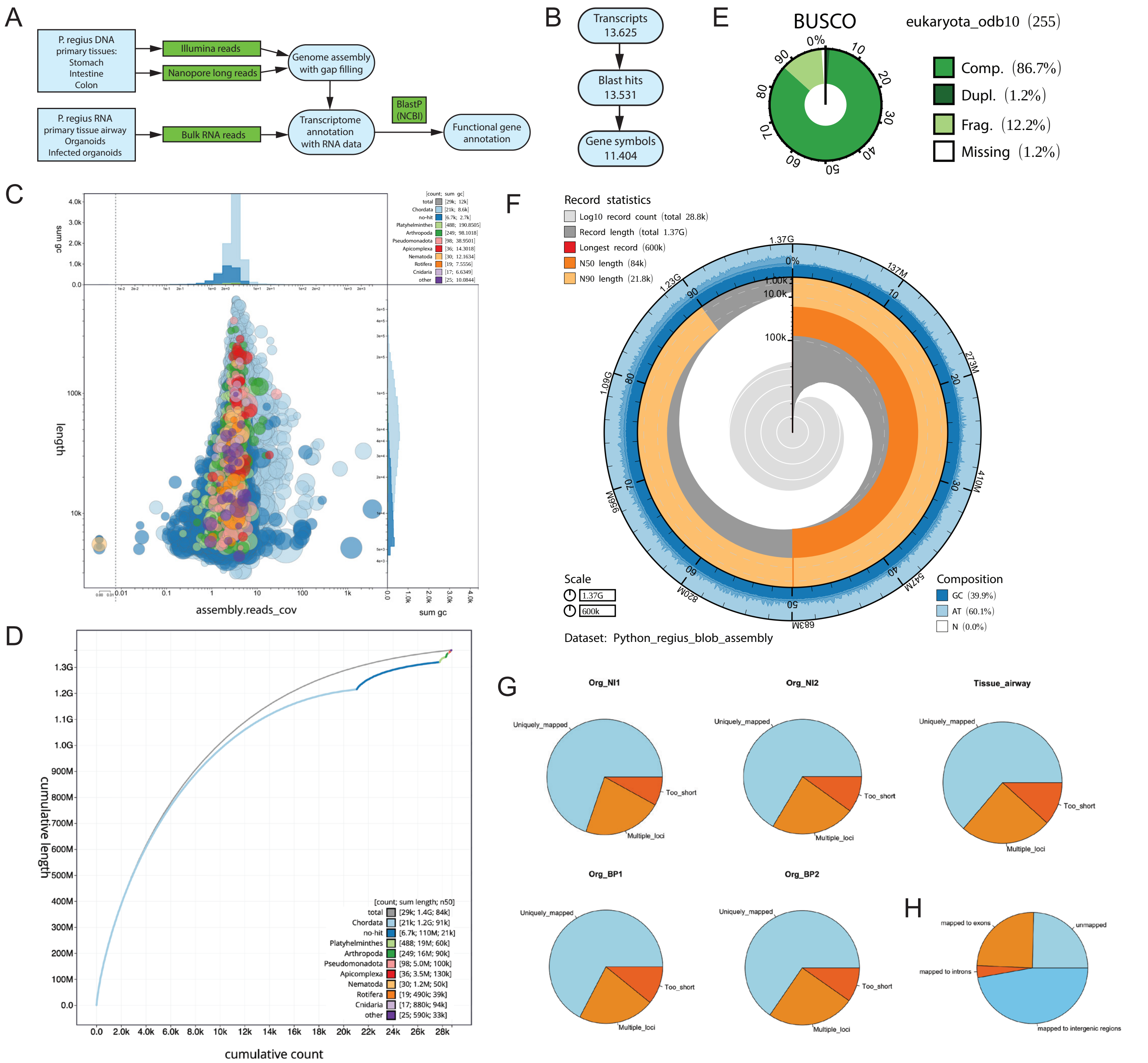


Supplementary Figure 1. **De novo assembly of P. regius reference genome.** A) Schematic representation of the workflow for reference genome assembly. Short and long DNA reads were used to assemble the genome sequence. Bulk RNA reads from P.regius airway primary tissue and organoids were used to annotate the transcriptome. B) Success rate of functional gene annotation for identified P. regius transcripts. C) Blob plot of length against base coverage of nanopore sequencing in assembly reads for sequences in the P. regius assembly. Sequences are coloured by phylum. Circles are sized in proportion to sequence GC proportion on a linear scale, ranging from 0.32 to 0.691. Histograms show the distribution of the sequence GC proportion sum along each axis. D) Cumulative sequence length for P. regius assembly. The grey line shows cumulative length for all sequences. Coloured lines show cumulative lengths of sequences assigned to each phylum using the bestsumorder taxrule and are stacked by cumulative value on the x-axis to show the proportion of each phylum in the overall assembly. E) Pie chart showing a summary of complete, fragmented, duplicated and missing BUSCO genes in the eukaryota_odb10 set. F) Snail plot summary of assembly statistics for P. regius assembly. The main plot is divided into 1,000 size-ordered bins around the circumference, with each bin representing 0.1% of the 1,366,273,982 bp assembly. The distribution of sequence lengths is shown in dark grey with the plot radius scaled to the longest sequence present in the assembly (599,941 bp). Orange and light-orange arcs show the N50 and N90 sequence lengths (84,023 and 21,812 bp), respectively. The light grey spiral shows the cumulative sequence count on a log scale with white scale lines showing successive orders of magnitude. The blue and light-blue area around the outside of the plot shows the distribution of GC, AT and N percentages in the same bins as the inner plot. G) STAR mapping rates of bulk RNA sequenced P. regius samples (NI = non-infected, BP = infected with BPNV) mapped to the P. regius assembly. H) Cellranger mapping rates of single cell RNA sequencing of P. regius organoids mapped to the P. regius assembly.

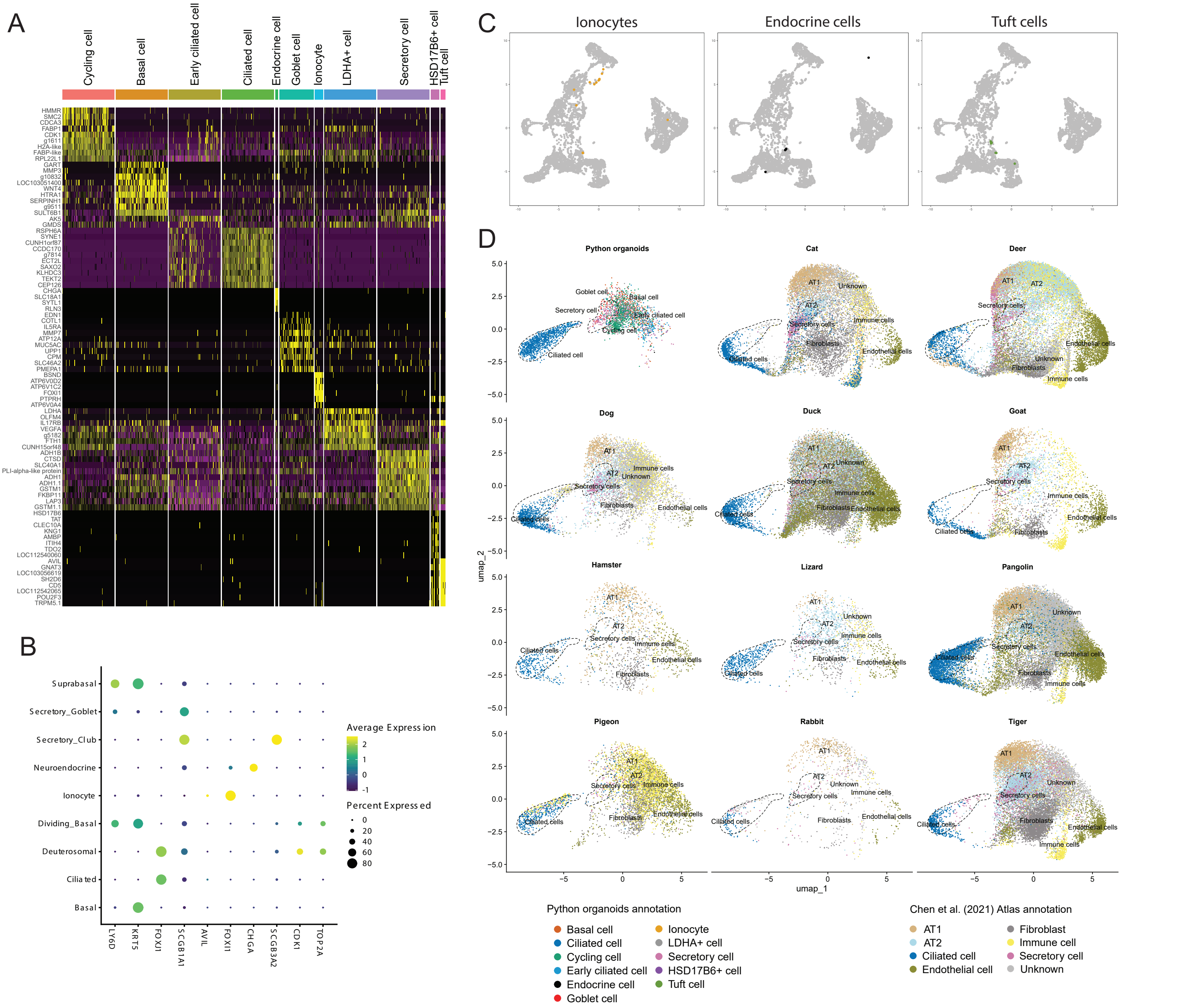

Supplementary Figure 2. **Extendend single cell sequencing analysis results.** A) Differential gene expression across P. regius cell type clusters. Shown are the top differentially expressed genes per cell type for 100 randomly sampled cells, or for all cells if fewer than 100 were available. B) Marker gene expression for epithelial cell types in the Madissoonn et al. primary human data set. C) UMAP projection of P. regius ionocytes (yellow), endocrine cells (black) and tuft cells (green) onto human primary data set (grey). D) Integration of P. regius airway organoid single cell sequencing with 11 non-model animals from the Chen et al. atlas. Dotted lines indicate the locations of P. regius ciliated and secretory cells within the UMAP projection.

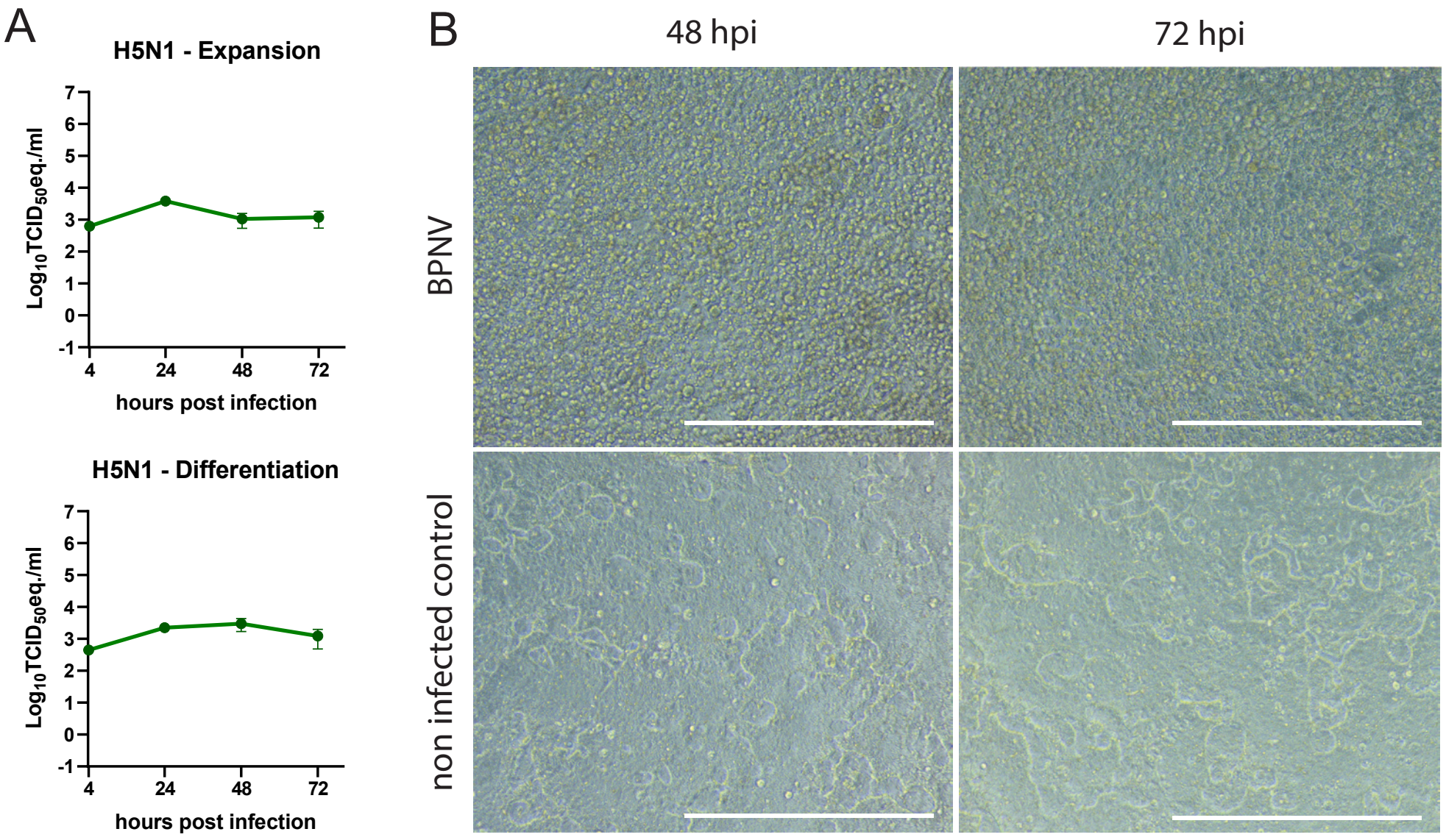


Supplementary Figure 3. **Infection of P. regius organoids.** A) Quantification of Influenza A genome levels in infected expansion (EM) or differentiated (DM) airway organoid monolayers. Data are presented as mean values +/- SD. B) Cytopathic effects of BPNV in airway organoid monolayers cultured in expansion medium at 48 and 72 hours post infection. Representative images of 2 independent experiments. Scale bar = 400 µm.
