# Supplementary material for: Modeling reptile virus infection in vitro using Python regius airway organoids
Source: Nat Commun. 2026 Aug 13;17:9739. doi: 10.1038/s41467-026-76411-9 (PMC13562644; doi:10.1038/s41467-026-76411-9)
Supplement: Supplementary file 3 [file 41467_2026_76411_MOESM5_ESM.pdf]

nature portfolio

Peer Review File

Modeling reptile virus infection in vitro using Python regius airway organoids

Corresponding Author: Professor Hans Clevers

This file contains all reviewer reports in order by version, followed by all author rebuttals in order by version.

Version 0:

Reviewer comments:

Reviewer #1

(Remarks to the Author)
In the manuscript entitled ‘Modelling reptile virus infection in vitro using Python regius airway organoids’, authors Willemsen et al. describe and establish for the first time a model of lung organoids in a reptile species as a model for studying the reservoir status of potentially zoonotic viruses. In addition, the approaches to transcriptomic characterisation and the study of the interaction of a Nidovirus – BPNV – with these organoids demonstrate the usefulness of developing these in vitro models for species that have been relatively little studied.
As a general comment, the manuscript is clear and remarkable in terms of the development of this original model for this species. Nevertheless, a few points, mainly technical, deserve attention and clarification and the entire paragraph on viral testing is very incomplete and too vague to allow for any conclusions to be drawn.
Line 270: What is the origin of the animal/egg – not specified. Is it of zoological origin? Is the species protected and what authorisation does this imply?
Line 285: Organoids in the expansion phase are maintained for more than 40 passages with a passage and ratio of 1/3, but are the passages in days regular? It would be desirable to establish a growth curve with the days and split ratios.
What are the cryopreservation conditions, in what volume and at what concentration?
Line 309: the value used is 0.1, but what does this correspond to if the total number of cells is not indicated? How many cells are there at confluence? It would be preferable to indicate the number of particles per well. What is the differentiation time used – d21? Why this choice? – Contradiction? with 16 days of differentiation (line 120)?
For the virus, on which cell type is the titration performed – id for line 331 – the titre is indeed very dependent on the cell type used. Is it not a reptile cell model?
Figure 4: Does the virus induce cell lysis? What measurement – impedance – is used to monitor the effect of infection? Idem for H5N1 in SD – which are not referenced in the text?? The images are illegible. An impedance measurement would be more relevant to demonstrate a cytotoxic effect and prove the reality of both BPNV and H5N1 infections – not convincing on the curves, because what is the initial load? Furthermore, what is the control on cell viability during treatment with Remdesivir for a possible toxic effect of the molecule? This entire “viral” paragraph is very poorly presented, incomplete and confusing.
Line 315: what is the origin of the antiviral molecules (reference?)
Lines 409-414: are the GEO references incomplete?
Figure 1 E: there is no visibility of the cells? What is the point? Phalloidin/DAPI staining to better see the cell boundaries and greater magnification?

Reviewer #2

(Remarks to the Author)

The study by Willemsen and colleagues presents an important and timely advance by establishing, to my knowledge, the first reptilian airway organoid model, derived from the ball python (Python regius). This work provides an important novel in vitro platform to study reptile-specific airway pathogens and respiratory disease processes in their natural target tissue, which was not previously possible in these non-model vertebrates. The authors combine organoid culture with single-cell RNA sequencing to generate a molecular cell atlas of the python airway epithelium, revealing a greater diversity of cell types than previously known, including rare populations such as endocrine cells, ionocytes, and tuft cells. This represents a

valuable contribution to our understanding of reptilian respiratory biology. A particular strength of the study is the use of these physiologically relevant airway organoids to evaluate antiviral drug efficacy. The authors demonstrate that Remdesivir is effective whereas Ribavirin is not, in contrast to earlier studies using non-target cell types. These findings highlight the importance of using tissue-specific infection models to accurately evaluate drug effects and emphasize the broader relevance of the organoid models presented in the study.

Overall, the study is novel, technically outstanding and potentially impactful. However, there are several substantive issues that should be addressed before the manuscript can be considered for publication.

1. The organoid culture is derived from a near-hatchling specimen, which may not be representative of adult airway biology

The organoid culture is well-established with histology and immunostaining and supported by extensive single-cell RNAseq characterization. The infection experiments show clear transcriptomic responses and the drug assay differentiates between the effects of Remdesivir and Ribavirin. Overall, these results provide a solid and comprehensive framework for studying reptilian airway biology and host-pathogen interactions. However, organoid development, single-cell RNAseq profiling, infection-response analyses and antiviral assays are all based on tissue obtained from a single, near-hatchling specimen, which may not reflect adult airway biology, immune competence or pharmacologic sensitivity. This limited biological sampling makes it difficult to extrapolate the findings to adult snake airway biology. Across vertebrates it is known that age-dependent epithelial maturation, immune pathways and barrier properties can influence cell-type composition, gene expression, antiviral responses and drug penetration, and thus, the reported cell-type proportions and other results such as drug efficacy may represent juvenile-specific features rather than species-level characteristics. While the technical depth of the study is outstanding, relying on a single pre-hatchling donor specimen might potentially introduce systematic bias, so authors should explicitly acknowledge this limitation and tone down species-level claims accordingly. I would also recommend to include a brief discussion about how age-related differences might affect interpretation of the results as I think it will greatly strengthen the manuscript.

2. Cell type identification is based on human annotations, without incorporating available single-cell datasets from closely related reptiles

Cell types are identified based on marker genes and cross-species projections and immune responses are interpreted using GO enrichment and curated host-defense gene sets. These analyses provide a combined overview of epithelial cell type diversity and antiviral responses in the python airway organoids. However, the molecular characterization relies mainly on human-based orthology mappings, human single-cell atlases and GO annotations applied to a custom Python regius genome with incomplete immune gene annotation, which might potentially introduce a systematic interpretive bias. Human cell atlas projections may misassign reptile-specific or lineage-divergent cell states and human GO categories can underestimate or distort pathway representation when key genes are missing or poorly annotated in the reference genome. These issues are relevant specially for the identification of rare cell populations in Figure 2 and for the interpretations about the breadth and composition of antiviral signaling pathways in Figure 3. Authors should explicitly acknowledge these limitations and modify the main claim to reflect this nuanced perspective. I would recommend that authors integrate and cite available single-cell datasets from non-model reptiles and other amniotes, which would provide a more appropriate comparative framework. Specifically, authors should include and discuss the study by Chen et al (2021) since it would strengthen cell-type annotations and help place them in a more phylogenetically relevant context, reducing reliance on human-based annotations.

Chen, D., Sun, J., Zhu, J. et al. Single cell atlas for 11 non-model mammals, reptiles and birds. Nat Commun 12, 7083 (2021). https://doi.org/10.1038/s41467-021-27162-2
https://www.nature.com/articles/s41467-021-27162-2

3. Absent genome assembly and quality metrics for the Python regius reference genome assembled to enable molecular characterization of organoids

The authors generate a Python regius reference genome using a hybrid assembly of Illumina and Nanopore reads, with a length of 1.36 GB and 11,404 annotated genes. This reference genome and its annotation are the basis for mapping and interpreting single-cell and bulk RNA-seq from the python airway organoids. This genome is a valuable resource that supports the molecular analyses presented, however, the study does not report standard genome assembly and annotation quality metrics (e.g., contig/scaffold N50, BUSCO completeness, read mapping rates, or contamination screening). Without these metrics, the quality and reliability of the reported genome cannot be evaluated, and it is unclear if missing pathways (e.g., type I/II interferons) reflect true biological absence or assembly/annotation incompleteness. Given that downstream analyses in the single-cell and infection-response datasets are based on this genome, lack of completeness and contamination would directly affect confidence in the gene identities and pathway coverage. Authors should report assembly quality metrics, including contig/scaffold N50/L50 and GC content (e.g., via QUAST), as well as Illumina and Nanopore read mapping rates (BWA-MEM2 and minimap2). Assembly completeness should be assessed with BUSCO and k-mer–based metrics (e.g., Merqury), and potential contamination screened with BlobToolKit. Targeted TBLASTN searches for interferon and TLR families, followed by domain annotation, would help clarify whether apparently missing immune genes are true biological absences or due to technical issues. Reporting scRNA-seq and bulk RNA-seq mapping rates to the reference genome would also be desirable. These data would provide quantitative support for assembly quality and clarify the

reliability of downstream results.

4. The antiviral experiments are based on only two replicates, which is insufficient to demonstrate the physiological relevance of the organoid model for drug testing.

The antiviral drug experiments are interesting and the differences observed between Remdesivir and Ribavirin are potentially relevant. However, the statistical analysis is based on only two technical replicates per condition, which is insufficient to support meaningful conclusions. With such limited replication, the physiological relevance of the organoid model for drug testing is not convincingly demonstrated. The results should therefore be interpreted with caution and considered only exploratory. The manuscript should clearly state that the conclusions are limited by the low number of replicates. In addition, I would recommend to include a power analysis to estimate the number of biological replicates required to achieve adequate statistical power (e.g., 80%). If possible, the authors should also report the estimated statistical power associated with the current sample size and experimental design.

Minor comments

- Please use the abbreviations EM and DM consistently in both the main text and the figures and keep the nomenclature uniform across the manuscript.

- Use the abbreviation Ball Python Nidovirus (BPNV) only after the first definition and then consistently throughout the manuscript; alternatively, it may be clearer to avoid the abbreviation.

- Line 135: Change “yes” for “yet”.

- Line 164: I couldn’t find general information on the bulk RNA analysis. This should be included, at least in the Supplementary Materials, providing the overall data or summary of the bulk analysis.

- Line 260: Make sure P. regius is written correctly and consistently throughout the manuscript.

- Line 336: Is the staining phalloidin or F-actin? If it is phalloidin, as stated in the Methods, please revise Figure 1C accordingly.

- Figure 1A: Change “Python Regius” for “Python regius”

- Figure 1C: DAPI should be shown in orange and F-actin (or phalloidin) in blue.

- Figure 3A: Is the stained dsRNA in infected cells shown in red? If so, please specify this in the figure legend.

- Figure 3B and Supplementary Figure 3A (line 160-162): The difference between the two conditions is not very clear from the current presentation. For example, in Fig. 3B at 72 hpi both values appear to be around 4. At first glance, the values seem quite similar, which makes it difficult to appreciate the statement that the levels are higher in EM. It might be clearer to plot both conditions in the same graph for direct comparison, or at least to provide the numerical values for each data point. Including these values, either in the figure or the text, would help the reader better understand the differences.

- Figure 3E: Change “upregulated of genes” for “upregulated genes” or “upregulation of genes”.

Reviewer #3

(Remarks to the Author)
I co-reviewed this manuscript with one of the reviewers who provided the listed reports. This is part of the Nature Communications initiative to facilitate training in peer review and to provide appropriate recognition for Early Career Researchers who co-review manuscripts.

Version 1:

Reviewer comments:

Reviewer #1

(Remarks to the Author)
The authors have taken most of the comments on board and provided the necessary information to address the points raised.
But just one important detail: when the response states, ‘However, we have not kept detailed records of the timing of the passaging’, this is surely implausible – or is there simply no record of the experiments carried out???
If there are no data on this cell culture base – the entire foundation of the work – then how much credibility can be given to all

the other results – which are also not recorded?
It is reckless to put forward this argument!

Reviewer #2

(Remarks to the Author)
We thank the authors for their careful revision and for thoroughly addressing all comments and clarifying the issues raised during the review process. The manuscript has been substantially revised, and the authors have taken into account all requests, suggestions and recommendations, and we think it has strengthened their already great contribution.

Reviewer #3

(Remarks to the Author)
I co-reviewed this manuscript with one of the reviewers who provided the listed reports. This is part of the Nature Communications initiative to facilitate training in peer review and to provide appropriate recognition for Early Career Researchers who co-review manuscripts.

Open Access This Peer Review File is licensed under a Creative Commons Attribution 4.0 International License, which permits use, sharing, adaptation, distribution and reproduction in any medium or format, as long as you give appropriate credit to the original author(s) and the source, provide a link to the Creative Commons license, and indicate if changes were made.
In cases where reviewers are anonymous, credit should be given to 'Anonymous Referee' and the source.
The images or other third party material in this Peer Review File are included in the article's Creative Commons license, unless indicated otherwise in a credit line to the material. If material is not included in the article's Creative Commons license and your intended use is not permitted by statutory regulation or exceeds the permitted use, you will need to obtain permission directly from the copyright holder.
To view a copy of this license, visit https://creativecommons.org/licenses/by/4.0/
