# Supplementary material for: Modeling reptile virus infection in vitro using Python regius airway organoids
Source: Nat Commun. 2026 Aug 13;17:9739. doi: 10.1038/s41467-026-76411-9 (PMC13562644; doi:10.1038/s41467-026-76411-9)
Supplement: Supplementary file 4 [file 41467_2026_76411_MOESM1_ESM.docx]

Title: Supplementary data 1

Description: BlastP annotation hits for identified gene transcripts.
